# COGNITIVE AND HEARING IMPAIRMENTS IN OLDER ADULTS: EVIDENCE FROM THE HEALTH AND RETIREMENT STUDY

**DOI:** 10.1093/geroni/igz038.300

**Published:** 2019-11-08

**Authors:** Jessica S West, Scott Lynch

**Affiliations:** Duke University, Durham, North Carolina, United States

## Abstract

As the number of older adults increases, increased prevalence of cognitive and sensory impairments pose growing public health challenges. Research on the relationship between hearing impairment and cognition, however, is minimal and has yielded mixed results, with some studies finding that hearing impairment is associated with cognitive decline, and others reporting that the association is weak or non-existent. Most of this research has been conducted outside of the U.S., and the few U.S.-based longitudinal studies have relied mostly on small, non-representative samples involving short follow-up periods. Further, despite known gendered patterns in cognitive and hearing impairments, no studies to date have examined whether the relationship between the two varies by gender. Our study addresses these weaknesses in the literature by utilizing nine waves of the Health and Retirement Study (1998-2014; n=14,169), a large, nationally representative, longitudinal study that facilitates examination of long-term interrelationships between hearing and cognitive impairments. In this study, we use autoregressive latent trajectory (ALT) methods to model: 1) the relationship between hearing impairment and cognitive decline, and 2) sex differences in the relationship. ALT models enable us to determine whether hearing impairment and cognitive impairment are associated, net of their common tendency simply to co-trend with age. Results indicate that hearing and cognitive impairments are strongly interrelated processes that trend together over time. Moreover, hearing impairment has an increasing impact on cognitive impairment across age while the effect of cognitive impairment on hearing impairment levels out over time. Sex differences in these patterns are discussed.

